# Diagnostic Accuracy of the Diffusion-Weighted Imaging Method Used in Association With the Apparent Diffusion Coefficient for Differentiating Between Primary Central Nervous System Lymphoma and High-Grade Glioma: Systematic Review and Meta-Analysis

**DOI:** 10.3389/fneur.2022.882334

**Published:** 2022-06-24

**Authors:** Xiaoli Du, Yue He, Wei Lin

**Affiliations:** ^1^Department of Radiology, Chengdu First People's Hospital, Chengdu, China; ^2^Department of Orthopedics, Chengdu First People's Hospital, Chengdu, China

**Keywords:** diffusion-weighted imaging, meta-analysis, lymphoma, diagnosis, high-grade glioma

## Abstract

**Background:**

It is difficult to differentiate between a few primary central nervous system lymphoma (PCNSL) and high-grade glioma (HGG) using conventional magnetic resonance imaging techniques. The purpose of this study is to explore whether diffusion-weighted imaging (DWI) can be effectively used to differentiate between these two types of tumors by analyzing the apparent diffusion coefficient (ADC).

**Research Design and Methods:**

Data presented in Pubmed, Embase, Web of Science, Cochrane Library, China National Knowledge Infrastructure (CNKI), Wanfang Database, and China Science and Technology Journal Database (CQVIP) were analyzed. High-quality literature was included, and the quality was evaluated using the quality assessment of diagnostic accuracy studies-2 (QUADAS-2) tool, and the studies were based on the inclusion and exclusion rules. The pooled sensitivity, pooled specificity, pooled positive likelihood ratio (PLR), pooled negative likelihood ratio (NLR), pooled diagnostic odds ratio (*DOR*), area under the curve (AUC) of the summary operating characteristic curve (SROC), and corresponding 95% confidence interval (*CI*) were calculated using the bivariate mixed effect model. Meta-regression analysis and subgroup analysis were used to explore the sources of heterogeneity. The publication bias was evaluated by conducting Deek's test.

**Results:**

In total, eighteen high-quality studies were included. The pooled sensitivity was 0.82 (95% CI: 0.75–0.88), the pooled specificity was 0.87 (95% CI: 0.84–0.90), the pooled positive likelihood ratio was 6.49 (95% CI: 5.06–8.32), the pooled NLR was 0.21 (95% CI: 0.14–0.30), the pooled *DOR* was 31.31 (95% CI: 18.55–52.86), and the pooled AUC was 0.90 (95% CI: 0.87–0.92). Sample size, language and country of publication, magnetic field strength, region of interest (ROI), and cut-off values of different types of ADC can potentially be the sources of heterogeneity. There was no publication bias in this meta-analysis.

**Conclusions:**

The results obtained from the meta-analysis suggest that DWI is characterized by high diagnostic accuracy and thus can be effectively used for differentiating between PCNSL and HGG.

## Introduction

Primary central nervous system lymphoma (PCNSL) is a variant of the non-Hodgkin lymphoma of the central nervous system. PCNSL is a disease in which cancer cells form in the primary brain, spinal cord, and eye (1). The origin of 3–5% of intracranial tumors can be attributed to PCNSL. It has been observed that the incidence rate of PCNSL is increasing each year (2). The clinical manifestations are similar to the clinical manifestations observed for intracranial gliomas. Apart from the process of pathological biopsy, imaging techniques are also used to differentiate between the two types of tumors. At present, the recommended PCNSL treatment method involves the use of chemotherapy combined with immunotherapy. Generally, surgery is not recommended (3, 4). HGG is primarily resected surgically, and the process is supplemented by radiotherapy and chemotherapy (5). The choice of the treatment plan heavily relies on the accurate identification of the two types of tumors.

A significant number of uniformly enhanced masses or nodules are observed in the deep parts of the brain near the midline structure when the MRI technique is used to study the manifestation of PCNSL. Atypical MRI manifestations, which overlap with the manifestations observed for HGG, are observed in some cases, making the accurate identification of the tumors difficult. This can potentially result in misdiagnosis. Diffusion-weighted imaging (DWI) is a new MRI technology that was developed in the middle of the 1990s. It is the only non-invasive method that can be used to detect the diffusion of water molecules in living tissues (6). DWI does not involve the process of contrast agent imaging. This process can be used to diagnose patients allergic to contrast agents or patients suffering from abnormal renal function. The results obtained using this technique are better than the result obtained using other techniques, such as enhanced MRI. The apparent diffusion coefficient (ADC) value can eliminate the effects of the T2 penetration effect and diffusion-sensitive gradient. This value can accurately and truly reflect the diffusion ability of water molecules in tumor tissues. The ADC value can quantitatively reflect the density and malignancy of tumor cells. Hence, it is widely used in clinical diagnosis (7). We hypothesized that the ADC value could be used to distinguish between the two types of tumors as the extents of blood flow characterizing PCNSL and HGG are different. Based on the current research status, we conducted a systematic meta-analysis to evaluate the comprehensive diagnostic value of DWI combined with ADC for accurately identifying PCNSL and HGG.

## Materials and Methods

### Search Strategy

Chinese and English databases were primarily searched. The English databases include Pubmed, Embase, Web of Science, and Cochrane Library, and the Chinese databases include China National Knowledge Infrastructure (CNKI), Wanfang Database, and China Science and Technology Journal Database (CQVIP). The keywords used were glioma, lymphoma, DWI, and ADC. We can obtain the relevant “Medical Subject Headings” from https://www.ncbi.nlm.nih.gov/mesh/?term=. For different databases, different Boolean logic retrieval formulae were used. The period for database retrieval covered the period from the establishment of each retrieval database to March 2022.

### Inclusion and Exclusion Criteria

There were several inclusion criteria: 1) The patient should have pathologically confirmed PCNSL and high-grade glioma (the WHO grade III or grade IV). 2) The patient should not have been exposed to any treatment methods prior to being subjected to the imaging conditions. 3) The patient should have undergone DWI–MRI sequence tests. 4) Studies with sufficient data to calculate true-positive (TP), false-positive (FP), false-negative (FN), and true-negative (TN) values should be considered. For example, the considered for the studies contains raw data for all patients. The reports present the sensitivity and specificity achieved using DWI during the diagnosis of the two diseases. Sensitivity is expressed as TP/(TP+FN), and specificity is expressed as TN/(TN+FP). These formulae can be used to obtain the relevant data (4) The sample sizes greater than 10 should be considered. The were several exclusion criteria applied. 1) Cases, where animal experiments or other basic experiments were conducted, were not considered. 2) Data from non-original research articles, such as the data presented in review articles, conference reports, abstracts, comments, letters, and case reports were excluded. 3) Patients from the same cohort were not considered. When the research samples are from the same cohort, we consciously consider and choose the latest study reported or the study with the largest sample size.

### Data Collection and Quality Assessment

During the process, two researchers are selected. If there are differences among the authors, the differences are resolved through discussion within the group. The extraction parameters primarily include the author of the study, the year of publication, the sample size (control group and case group sizes), the average age and gender of the patients in the control and case groups, research type (whether it is a retrospective study or a prospective study), imaging parameters corresponding to DWI, sensitivity, specificity, cut-off value, and the TP, FP, TN, and FN values of each study.

The quality of the included literature reports was evaluated based on the guidelines presented by the quality assessment of diagnostic accuracy studies-2 (QUADAS-2) tool and a Newcastle-Ottawa Scale (NOS). The QUADAS-2 scale was divided into various factors: 1) patient selection; 2) index test; 3) reference standard; and 4) flow and timing (8). The software Review Manager (version 5.2) was used to determine the bias and quality of the included studies. The literature quality evaluation chart was also drawn. The answers “yes,” “unclear,” and “no” corresponded to “high risk,” “unclear risk,” or “low risk,” respectively, and indicated the bias risk level. The risk levels were denoted in red, yellow, and green, respectively, in the quality evaluation chart. The NOS was used to assess the quality of included case-control and cohort studies and each item of NOS is assigned 1 or 2 points. Studies with a score of 5 or more are considered to be of high quality.

### Statistical Analysis

The data were analyzed using Stata 15.1 (64-bit), Review manager 5.2, and MetaDiSc 1.4. The pooled sensitivity, pooled specificity, pooled positive likelihood ratio (PLR), pooled negative likelihood ratio (NLR), pooled diagnostic odds ratio (*DOR*), summary receiver operating characteristic (SROC), area under the curve (AUC), and corresponding 95% confidence interval (*CI*) of DWI for the differentiation of PCNSL and HGG were calculated using the bivariate mixed effect model (9). The relationships among prior-test probability, likelihood ratio, and post-test probability were studied to certify using Fagan's nomogram. The heterogeneity of the study was assessed by conducting the Cochran-Q and *I*^2^ tests (10). A *p* < 0.05 was considered statistically significant. When *I*^2^ was > 50%, high heterogeneity among the studies was suggested. MetaDiSc 1.4 software was used to calculate the Spearman correlation coefficient between the logarithm of sensitivity and the logarithm of 1-specificity to determine whether the threshold effect results in heterogeneity. A strong positive correlation indicates the generation of a threshold effect (11). The literature publication bias was evaluated by analyzing the Deeks' funnel plot (12). Sensitivity analysis, meta-regression analysis, and subgroup analysis methods were used to identify the source of heterogeneity. The value of *p* < 0.05 was considered statistically significant.

## Results

### Literature Search and Quality Evaluation

**Figure 1:**
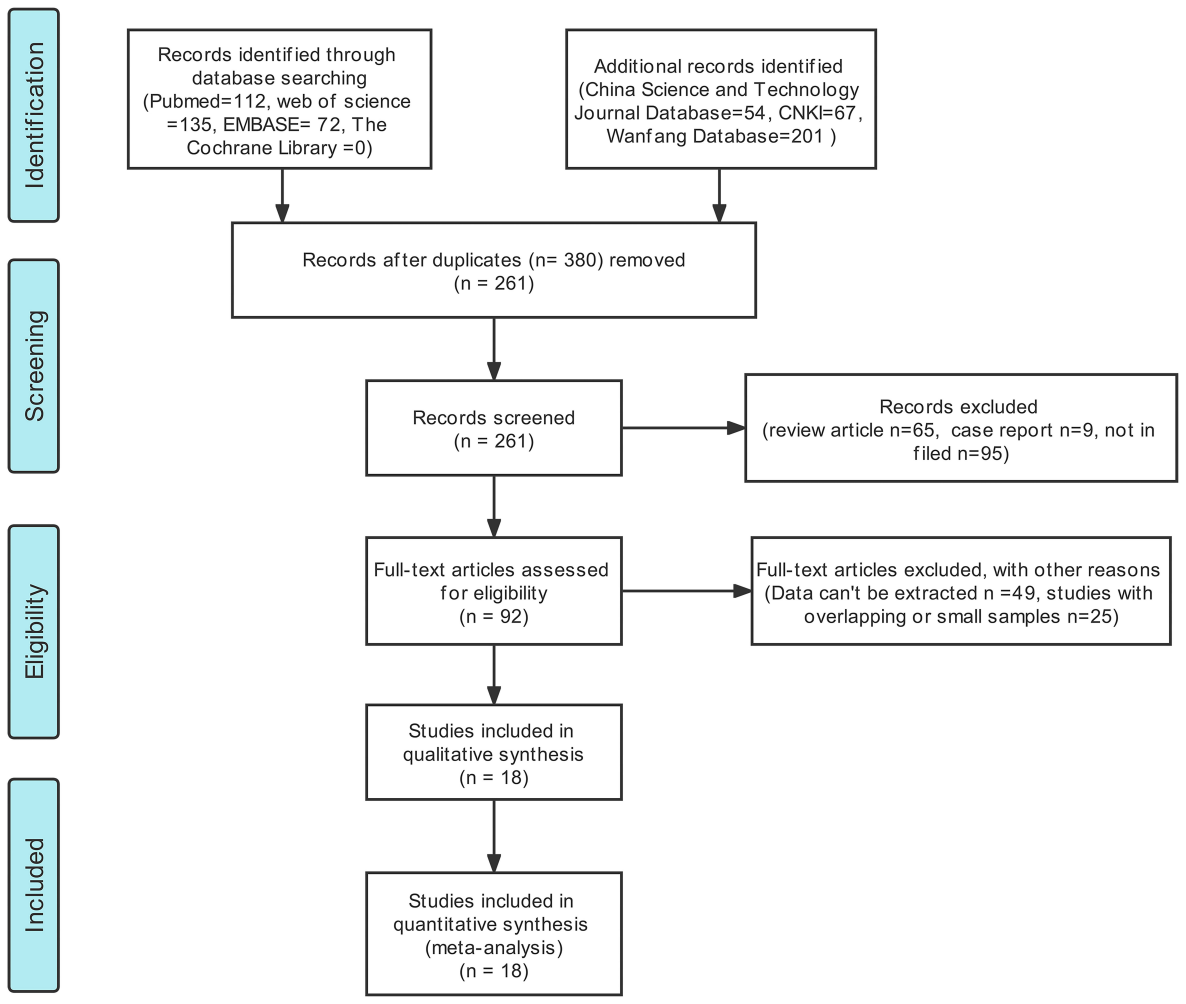
A Flowchart representing the literature selection process.

**Figure 2:**
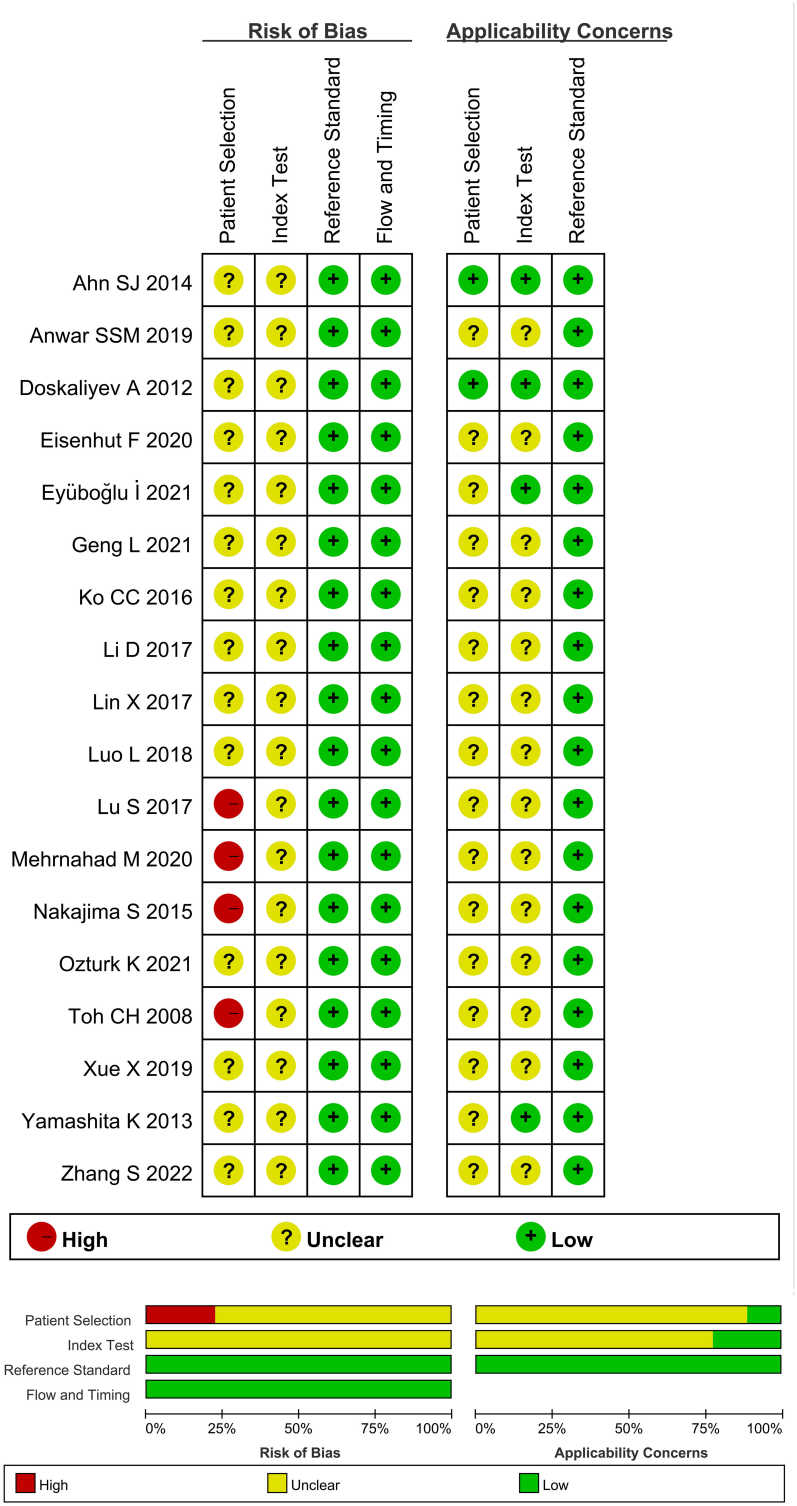
A document quality evaluation chart prepared using the QUADAS-2 tool.

We have preliminarily identified 261 documents (duplicate documents were excluded) for our studies by searching the Chinese and English databases. After reading the abstract and judging the literature type, we excluded 169 non-original studies (literature and review articles unrelated to this field of study). After thoroughly reading the remaining literature reports, we included a total of 18 literature reports (13–30) for meta-analysis. A total of 372 patients with PCNSL and 701 patients with glioma were included in the study. A total of 32 lesions (14 glioblastomas and 18 PCNSL) were studied by Anwar et al. (24). Hence, a total of 380 lesions characterized by PCNSL and 704 lesions characterized by glioma were included in the meta-analysis. The process of literature screening is presented in Figure 1, and the basic characteristics of the included literature reports are shown in Tables 1, 2. Results obtained from QUADAS-2 are presented in Figure 2, and the quality evaluation of NOS are shown in Supplementary Table 1. The quality of the included studies was found to be satisfactory.

**Table 1:** Basic clinical characteristics and parameters of the included literature.

**First author**	**Year**	**Language**	**Country**	**Study design**	**Type of magnetic resonance equipment**	**B values (s/mm^**2**^)**	**PCNSL**			**HGG**		
							**No. of patients**	**Mean age**	**SEX (Male/** **Female)**	**No. of patients**	**Mean age**	**SEX (Male/** **Female)**
Toh CH	2008	English	China	Retrospective	Siemens 3.0T	0, 1,000	10	53.3	4/6	10	51.9	5/5
Doskaliyev A	2012	English	Japan	Retrospective	GE 3.0T	0, 1,000, 4,000	10	59.7	4/6	14	60.2	7/7
Yamashita K	2013	English	Japan	Retrospective	Philips 3.0T	0, 1,000	19	64.8	Unknown	37	58.5	Unknown
Ahn SJ	2014	English	Korea	Retrospective	Philips 3.0T	0, 1,000	25	60	10/15	62	56.7	34/28
Nakajima S	2015	English	Japan	Retrospective	Siemens 3.0T	0, 1,000	11	70	4/7	23	56.5	13/10
Ko CC	2016	English	China	Retrospective	Siemens 1.5T	0, 1,000	22	59	9/13	104	60	58/46
Li D	2017	Chinese	China	Retrospective	Siemens 3.0T	0, 1,000	22	55	14/8	27	51	19/8
Lin X	2017	English	USA	Retrospective	GE 3.0T	1,000	18	68.7	11/7	36	68.6	22/4
Lu S	2017	English	China	Retrospective	Siemens 3.0T	0, 1,000	18	56.8	12/6	42	54.5	27/15
Luo L	2018	Chinese	China	Retrospective	Siemens 3.0T	0, 1,000	38	57	17/21	30	51	17/13
Xue X	2019	Chinese	China	Retrospective	GE 1.5T or 3.0T	0, 1,000	15	52.53	8/7	26	60.65	15/11
Anwar SSM	2019	English	Pakistan	Retrospective	Siemens 1.5T or 3.0T	0, 500, 1,000	10 (14)	56.8	3/7	11 (18)	52.0	8/3
Eisenhut F	2020	English	Germany	Retrospective	Siemens 3.0T	0, 1,000	37	68.7	24/13	37	67.9	25/12
Mehrnahad M	2020	English	Iran	Retrospective	Siemens 1.5T	0, 1,000	20	57	Unknown	50	54	Unknown
Geng L	2021	Chinese	China	Retrospective	GE 3.0T	0, 1,000	30	59.13	20/10	35	50.6	23/12
Eyüboglu I	2021	English	Turkey	Retrospective	Siemens 1.5T	0, 1,000	16	Unknowm	Unknown	55	Unknowm	Unknown
Ozturk K	2021	English	USA	Retrospective	Siemens 3.0T	0, 1,000	31	64	18/13	57	59	35/22
Zhang S	2022	English	China	Retrospective	GE 3.0T	0, 1,000	20	60.8	8/12	45	55.4	29/16

**Table 2:** Characteristic of primary diagnostic studies.

**First author**	**Year**	**Tp**	**Fp**	**Fn**	**Tn**	**Sensitivity**	**Specificity**	**Cut-off value**	**ROI placement**
Toh CH	2008	10	0	0	10	100	100	ADC ratios 1.060	Solid portion
Doskaliyev A	2012	9	1	1	13	90.9	91.7	ADC_min_ 0.5*10^−3^mm^2^/s	Solid portion
Yamashita K	2013	11	5	8	32	58.8	86.5	ADC_min_ 0.62*10^−3^mm^2^/s	Solid portion
Ahn SJ	2014	21	6	4	56	85	90	ADC_mean_ 0.98*10^−3^mm^2^/s	Whole tumor
Nakajima S	2015	11	6	0	17	100	73.9	ADC_5%_ 0.68*10^−3^mm^2^/s	Whole tumor
Ko CC	2016	14	17	8	87	64	84	ADC_t_ 0.77*10^−3^mm^2^/s	Solid portion
Li D	2017	21	4	1	23	95.5	85.2	ADC_c_ 0.77*10^−3^mm^2^/s	Whole tumor
Lin X	2017	12	4	6	32	69	89	ADC_mean_ 1.3*10^−3^mm^2^/s	Whole tumor
Lu S	2017	14	7	4	35	76.2	83.3	ADC ratios 1.317	Whole tumor
Luo L	2018	32	4	6	26	84.21	86.87	ADC_50%_ 1.1*10^−3^mm^2^/s	Whole tumor
Xue X	2019	10	0	5	26	66.7	100	ADC_mean_ 0.69*10^−3^mm^2^/s	Solid portion
Anwar SSM	2019	18	2	0	12	100	85.7	ADC ratios 1.05	Solid portion
Eisenhut F	2020	33	4	11	26	89	70	ADC_max_ 1.314*10^−3^mm^2^/s	Whole tumor
Mehrnahad M	2020	15	7	5	43	76	85	ADC_median_ 1.035*10^−3^mm^2^/s	Solid portion
Geng L	2021	29	2	1	33	96.7	95.7	ADC ratios 1.045	Whole tumor
Eyüboglu I	2021	12	12	4	43	76	78	ADC_tch_ 0.82*10^−3^mm^2^/s	Solid portion
Ozturk K	2021	23	7	8	50	73.7	87.1	ADC ratios 0.825	Whole tumor
Zhang S	2022	20	45	14	2	6	43	ADC_min_ 0.89*10^−3^mm^2^/s	Whole tumor

*ADC_min_, minimum apparent diffusion coefficient; ADC_meaan_, mean apparent diffusion coefficient; ADC_5%_, _fifth_ percentile value of cumulative apparent diffusion coefficient histogram; ADC_t_, apparent diffusion coefficient of the most strongly-enhanced tumor area; ADC_c_, corrected apparent diffusion coefficient; ADC_50%_, fiftieth percentile value of cumulative apparent diffusion coefficient histogram; ADC_tch_, apparent diffusion coefficient of tumor circumference hyperintensities*.

### Meta-Analysis: Results

**Figure 3:**
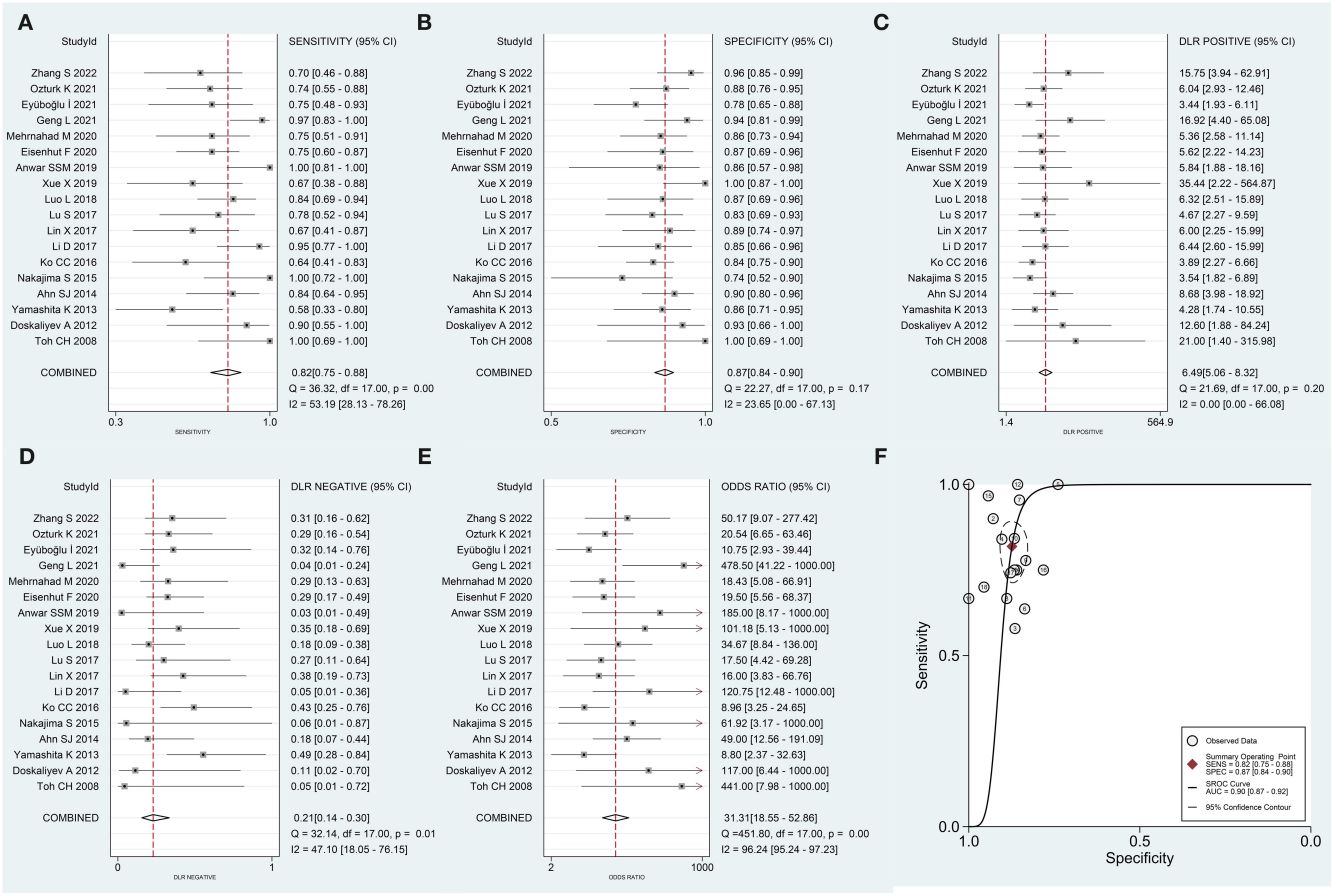
Forest plot generated for pooled **(A)** sensitivity, **(B)** specificity, **(C)** positive likelihood ratio (PLR), **(D)** negative likelihood ratio (NLR), **(E)** diagnostic odds ratio (DOR), and **(F)** area under the curve (AUC) recorded for the summary receiver operating characteristic (SROC) curve.

**Figure 4:**
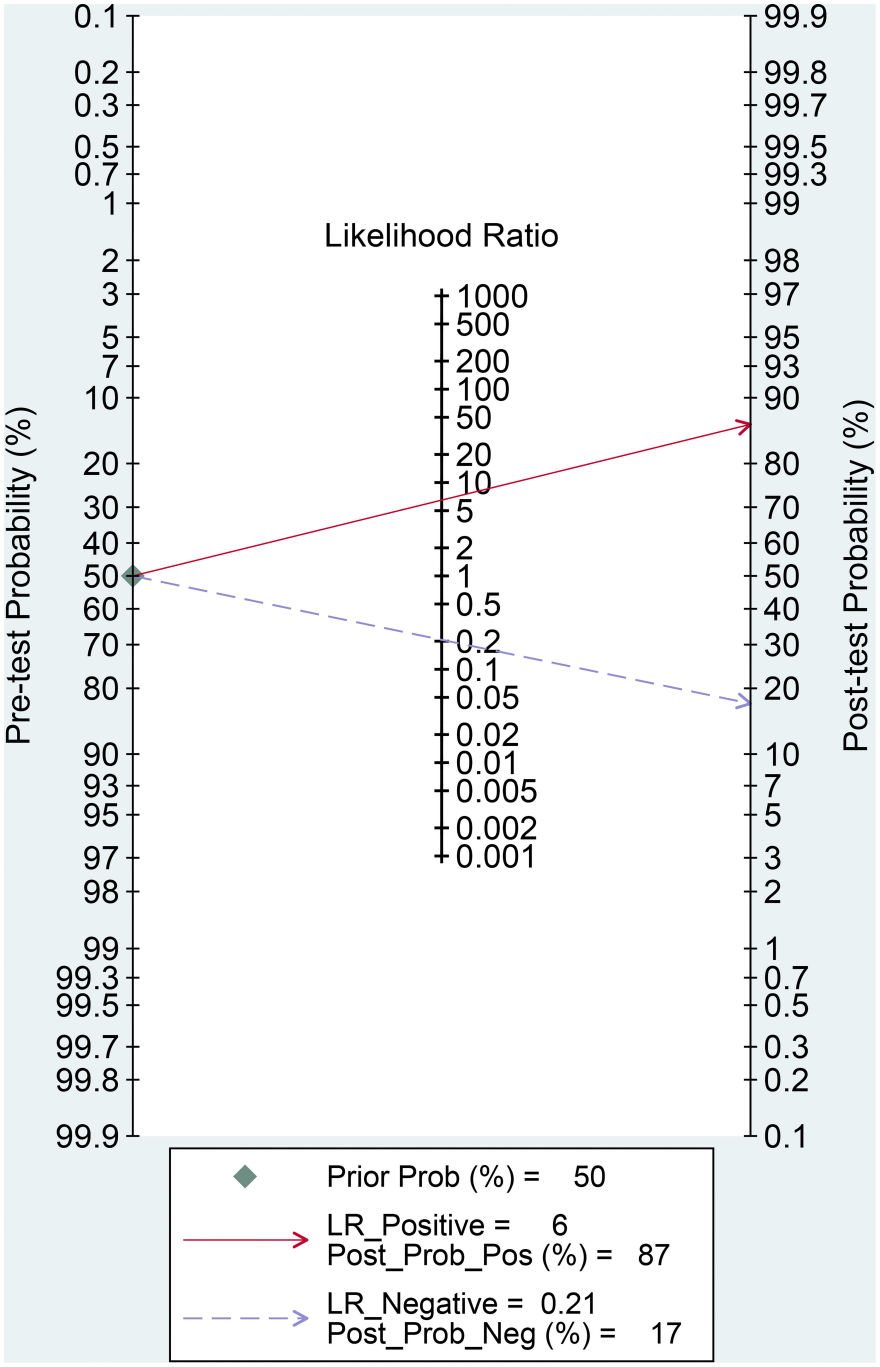
Fagan's nomogram for evaluating the post-test probability.

The results of the heterogeneity test were obtained (*Q* = 3.409, *df* = 2.00, *p* = 0.091, *I*^2^ = 41%; 95% *CI* was in the range of 0–100). It was observed that moderate levels of heterogeneity existed between the included studies. The results of the threshold effect test revealed that the Spearman correlation coefficient was 0.048 (*p* = 0.851). This indicated that a threshold effect did not result in heterogeneity. The pooled sensitivity of DWI (used to differentiate PCNSL from HGG) was 0.82 (95% CI: 0.75–0.88), the pooled specificity was 0.87 (95% CI: 0.84–0.90), the pooled PLR was 6.49 (95% CI: 5.06–8.32), the pooled NLR was 0.21 (95% CI: 0.14–0.30), and the pooled DOR was 31.31 (95% CI: 18.55–52.86). The forest maps of pooled results are shown in Figures 3A–E. The results obtained by analyzing the SROC are presented in Figure 3F. The AUC of SROC was 0.90 (95% CI 0.87–0.92). Analysis of the Fagan diagram (Figure 4) reveals that the probability before the prediction is 50%. Under conditions of positive DWI, the probability of diagnosing PCNSL would increase the post-test probability to 87%. When the result was negative, the probability of diagnosing PCNSL would decrease the post-test probability to 17%.

### Meta-Regression and Subgroup Analysis

**Figure 5:**
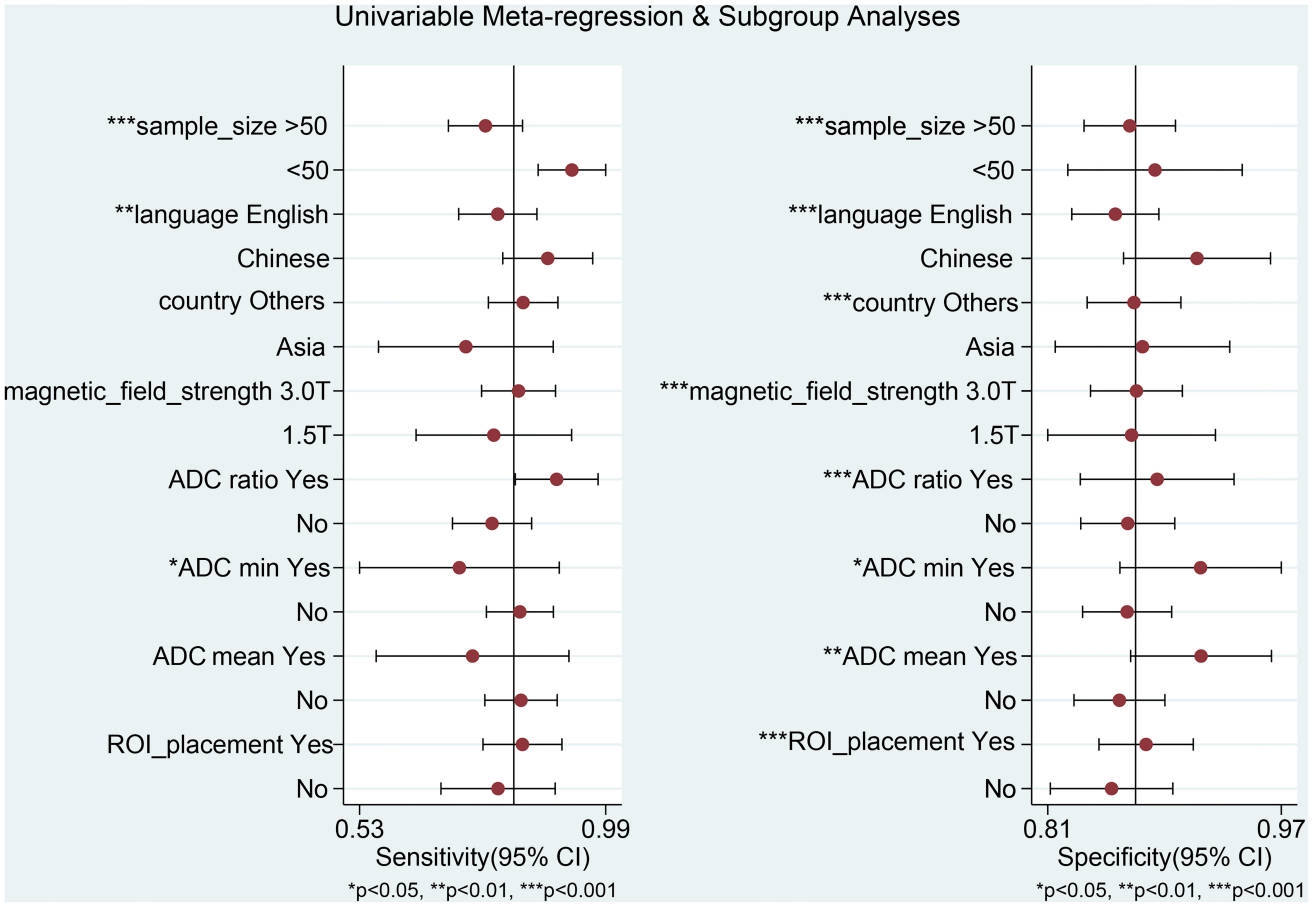
Univariable meta-regression analysis for the sensitivity and specificity of DWI for differentiating between primary central nervous system lymphoma (PCNSL) and high-grade glioma (HGG).

We conducted a meta-regression analysis to find the potential source of heterogeneity. Various variables were considered: sample size (>50 vs. <50), language (English vs. Chinese), country (Asia vs. other continents), magnetic field strength (3.0 vs. 1.5 T), the necessity of using ADC ratios, ADC_min_, and ADC_mean_ as the cut-off value (Yes vs. No), and ROI placement (solid portion vs. the whole tumor). This is presented in Figure 5. Some of the variables (sample size, language, and necessity of using ADC_min_ as the cut-off value) are the sources of heterogeneity for sensitivity and specificity, while others (country, magnetic field strength, necessity of using ADC ratios, ADC_mean_ as the cut-off values, and ROI placement) are the sources of heterogeneity observed in specificity.

For subgroup analysis (Table 3), the results of studies with a pooled literature whose sample size is less than 50 have a high sensitivity of 0.93 (95% CI: 0.86–0.99) and a specificity of 0.89 (95% CI: 0.83–0.95). Results from pooled studies conducted with Chinese language literature revealed that a high sensitivity of 0.88 (95% CI: 0.80–0.97) and a specificity of 0.92 (95% CI: 0.87–0.97) was achieved. Low sensitivity of 0.78 (95% CI: 0.70–0.85) was achieved in cases where ADC ratios were not used as the cut-off values. When ADC_min_ was not used as the cut-off value, high sensitivity of 0.83 (95% CI: 0.77–0.89) and low specificity of 0.87 (95% CI: 0.84–0.90) was achieved. In cases where ADC_mean_ was not used as the cut-off value, high sensitivity of 0.83 (95% CI: 0.76–0.90) and low specificity of 0.86 (95% CI: 0.83–0.89) were achieved. When ROI placement reflected a solid tumor, a low sensitivity of 0.79 (95% CI: 0.68–0.90) was achieved.

**Table 3:** Results of subgroup analysis and meta-regression analysis.

**Covariate**		**No. of studies**	**Sensitivity [95%CI]**	**P1**	**Specificity [95%CI]**	**P2**	**χ2**	***P***
Sample_size	>50	12	0.77 [0.70–0.83]	<0.01	0.87 [0.84–0.90]	<0.01	8.32	0.02
	<50	6	0.93 [0.86–0.99]		0.89 [0.83–0.95]			
Language	English	14	0.79 [0.72–0.86]	<0.01	0.86 [0.83–0.89]	<0.01	4.89	0.09
	Chinese	4	0.88 [0.80–0.97]		0.92 [0.87–0.97]			
Country	Asia	15	0.84 [0.77–0.90]	0.67	0.87 [0.84–0.90]	<0.01	1.82	0.40
	Others	3	0.73 [0.57–0.89]		0.88 [0.82–0.94]			
Magnetic field strength	3.0T	14	0.83 [0.76–0.90]	0.32	0.87 [0.84–0.91]	<0.01	0.36	0.83
	1.5T	4	0.78 [0.64–0.93]		0.87 [0.81–0.93]			
Parameter	ADC ratios	5	0.90 [0.82–0.97]	0.66	0.89 [0.84–0.94]	<0.01	4.40	0.11
	Others	13	0.78 [0.70–0.85]		0.87 [0.84–0.90]			
Parameter	ADC_min_	3	0.72 [0.53–0.90]	0.03	0.92 [0.86–0.97]	0.02	4.19	0.12
	Others	15	0.83 [0.77–0.89]		0.87 [0.84–0.90]			
Parameter	ADC_mean_	3	0.74 [0.56–0.92]	0.05	0.92 [0.87–0.97]	0.01	4.49	0.11
	Others	15	0.83 [0.76–0.90]		0.86 [0.83–0.89]			
ROI_placement	Whole tumor	10	0.83 [0.76–0.91]	0.13	0.88 [0.85–0.91]	<0.01	1.14	0.56
	Solid portion	8	0.79 [0.68–0.90]		0.86 [0.81–0.90]			

### Sensitivity Analysis and Publication Bias

**Figure 6:**
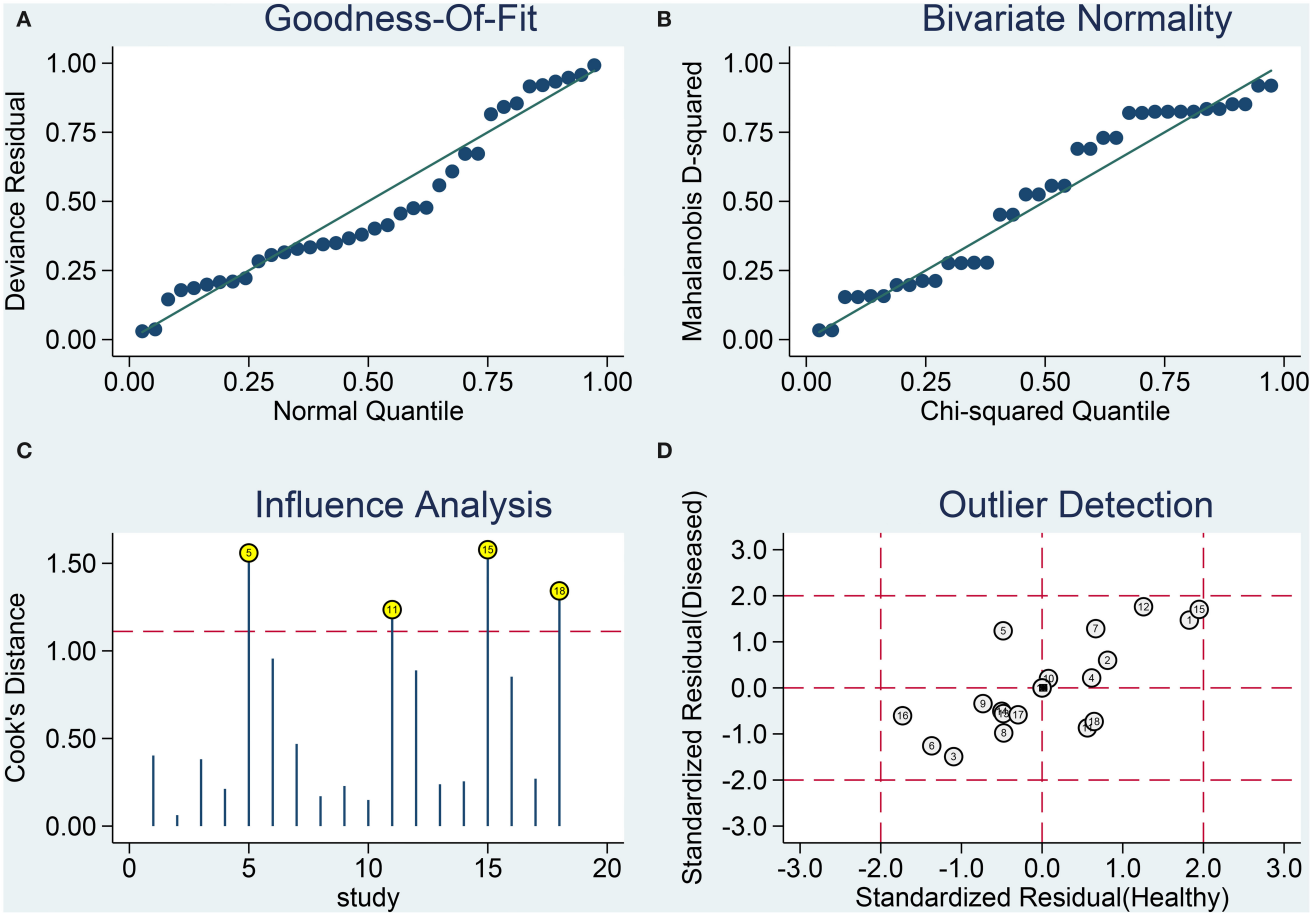
Results from sensitivity analysis. **(A)** Goodness-of-fit, **(B)** bivariate normality, **(C)** influence analysis, and **(D)** outlier detection.

**Figure 7:**
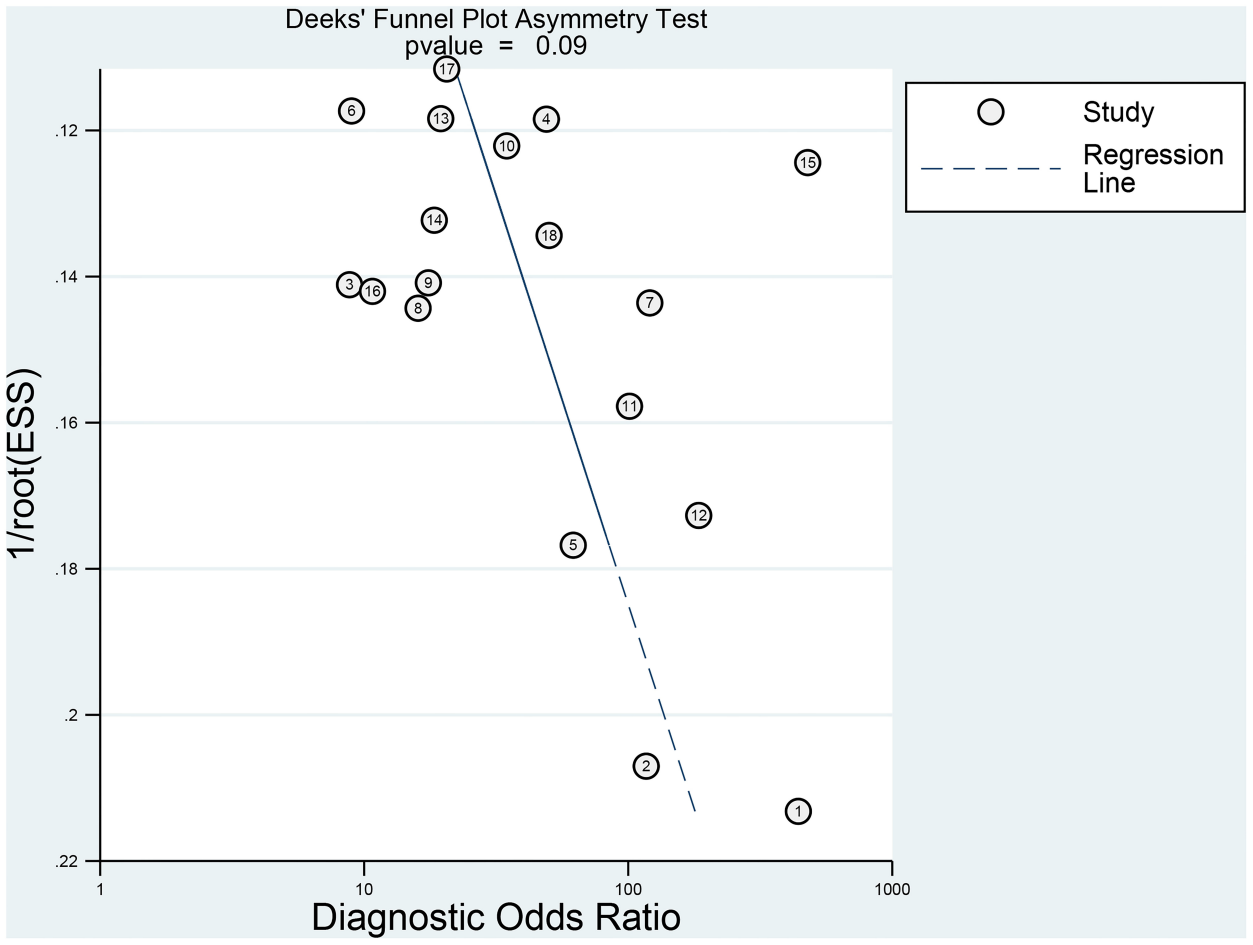
Publication bias determined by conducting Deeks' test.

Results from sensitivity analysis revealed that the goodness of fit and bivariate normality tests should be conducted using the bivariate mixed effect model for the meta-analysis (Figure 6). Four influential outliers were identified. No other outliers could be identified by conducting outlier detection tests. The four abnormal studies were excluded, and significant changes in sensitivity (0.83 vs. 0.82), specificity (0.85 vs. 0.87), PLR (5.65 vs. 6.49), NLR (0.20 vs. 0.21), AUC (0.86 vs. 0.90) and *DOR* (27.72 vs. 31.31) were not observed. This indicated that the combined results were relatively stable. Deek's test was performed to assess the publication bias (Figure 7). The number of studies on both sides of the dividing line was roughly equal and symmetrical (*p* = 0.09), indicating the absence of publication bias. Besides, the funnel plot was shown in Supplementary Figure 1.

## Discussion

We thoroughly searched and studied the Chinese and English databases to perform the meta-analyses. We selected a total of 18 relevant literature reports for the studies after thoroughly reading the literature reports (taking into account the inclusion and exclusion criteria). The pooled sensitivity, specificity, PLR, NLR, and DOR were 0.82, 0.87, 6.5, 0.21, and 31, respectively. The AUC was 0.90, indicating that DWI can effectively distinguish between PCNSL and HGG. The *DOR* is a single performance indicator that combines sensitivity and specificity for diagnostic testing. As the pooled *DOR* of the 18 studies selected by us was 31, it was advantageous to use DWI to distinguish PCNSL from HGG. Moderate levels of heterogeneity were obtained post meta-analysis. and there was no publication bias.

Primary central nervous system lymphoma and HGG are characterized by a similar location of onset, high cell density, multi-center onset, and invasive growth. Thus, there is a high chance of misdiagnosis (31). PCNSL can often be found in the deep brain parenchyma near the intracranial midline. It is mostly found on the tentorium. a slightly lower or equal T1 signal, equal or slightly higher T2 signal, highly intense DWI signal, low-intensity ADC signal, low incidence of internal cystic necrosis, mild to moderate peritumoral edema, and space-occupying effects can be observed using the MRI technique compared with other intracranial tumors (32). HGG invades a wide area (usually involving two or more lobes of the cerebral hemisphere). The invasion can be primarily observed in the white matter. Analysis of the images recorded using the MRI technique revealed the diffuse infiltration of the tumor cells and/or demyelination of the white matter. The signal was uniform, the boundary was unclear, and the tumor infiltration and edema could not be distinguished (33).

It has been previously reported that the density of the micro-vessel in PCNSL is significantly less than the density of the micro-vessels in HGG (34). This indicates that the tumor is deficient in blood. The rate of growth of these tumors is slow, resulting in less severe internal cystic necrosis. Glioma, especially HGG, is characterized by the high blood supply and rapid growth of tumor cells. The release of the cystic swelling factor (from the tumor cells) can result in a significant extent of cystic necrosis of the tumor tissues. The special anatomical position of some PCNSLs results in an adequate extent of blood supply. Under these conditions, the manifestations are complex and diverse. Hence, atypical MRI signals are obtained. The signals corresponding to PCNSL overlap with the atypical signals corresponding to glioma. Hence, it is difficult to accurately identify the two types of tumors using conventional MRI techniques. In recent years, the DWI method has been widely used in clinical settings as a non-invasive, rapid, and reproducible quantitative evaluation method to identify benign tumors, determine the degree of malignancy, and evaluate cell density.

Diffusion-weighted imaging is a non-invasive MRI-based functional imaging method that can be used to observe the microscopic dispersion state of water molecules present in tissues. The method can also be used for quantitative analysis in association with the ADC value. The ADC value can be used to eliminate the effects of the T2 penetration effect, diffusion sensitivity gradient, and other factors. This value efficiently and accurately quantifies the dispersion ability of the water molecules present in tumor tissues (35). ADC can quantitatively reflect the density and malignancy of tumor cells. In the past, most studies have used the average ADC of tumor parenchyma for comparison, but there is heterogeneity due to the different degrees of internal differentiation of tumor tissues. Only a single average ADC index cannot truly and accurately reflect the biological behavior and malignancy of tumors. In addition, results from the subgroup analysis also suggested that the sensitivity achieved using the ADC_min_ and ADC_mean_ values decreased significantly, while the sensitivity and specificity achieved using the ADC ratio increased. This further indicated that the ADC ratio could be effectively used to eliminate the influence of microcirculation injection and white matter fiber dispersion in different directions. It can also help increase the degree of comparability between different individuals and reduce systematic error. Therefore, a more comprehensive and effective performance should be achieved before the method is applied in clinical settings. A universal ADC value and specific ADC parameters to set the cut-off values are yet to be identified. The cut-off value for ADC varies from study to study. We should comprehensively analyze the images recorded for the patient to determine the specific ADC cut-off value. It has been reported that although large non-tumor parenchymal areas can be avoided to measure the average ADC values corresponding to tumor parenchyma, microcapsule formation, microcalcification, and bleeding in tumor parenchymal areas (potentially resulting in the deviation in the average ADC values) cannot be avoided in the region of interest. This hinders the reflection of tumor micropathology (28).

Previously reported results that were obtained by conducting meta-analyses have been combined and analyzed for the accurate identification of PCNSL and glioma using the MRI technique. For example, a meta-analysis conducted with 598 participants (pooled from 14 studies) revealed that the perfusion-weighted imaging (PWI) method could be effectively used to distinguish between HGG and PCNSL. The results were highly accurate (pooled AUC: 0.9415). The dynamic susceptibility contrast (DSC) value is potentially the best index that can be used to distinguish between HGG and PCNSL (36). Results from a meta-analysis involving 704 participants (pooled over 13 studies) revealed the absence of a significant difference in AUC [between DSC and susceptibility-weighted imaging (SWI)]. The Z-test was conducted, and the combined results suggested that a high and similar rate of diagnostic accuracy could be achieved using the DSC-MRI and SWI techniques. Thus, these methods could be effectively used to distinguish between HGG and PCNSL (37). Researchers have also conducted meta-analyses using the DWI technique to identify the two types of tumors. All eight studies were conducted on a total of 461 patients. The results obtained confirmed that the DWI method could be effectively used to accurately identify the two types of tumors. However, significant heterogeneity was observed, and the combined sensitivity, specificity, and other indicators were lower than those reported by us (38).

The ^18^F-fluorodeoxyglucose (FDG)-positron emission tomography/computed tomography (PET/CT) technique can be used to simultaneously reflect the anatomical and metabolic information of the focus. The use of the PET/CT technique can result in a significant increase in the diagnostic accuracy achieved for the central nervous system lymphoma (39). Uchinomura S et al. retrospectively analyzed the PET/CT scanning results of 13 patients with PCNSL and 62 patients with glioblastoma before they were subjected to treatment methods. It was found that the diagnostic AUC value of PETCT could be as high as 0.9 (39). A retrospective analysis was carried out by Zhou W et al. They reported that the maximum standardized uptake (SUVmax) value and tumor to normal contralateral cortex activity (T/N) ratio calculated using the PET/CT technique could be used as reliable indices to distinguish between PCNSL and glioblastoma multiforme (40). Currently, an increasing number of studies are being conducted using the machine learning (ML) technique to image brains and distinguish between PCNSL and HGG. Results obtained from a meta-analysis of 8 relevant literature reports suggest that the efficiency of the ML algorithm-based method was at par, or in some cases, better than the efficiency of radiologists. The pooled AUC was approximately 0.9 (41).

There are several limitations of the studies. First, the sample sizes in some of the literature reports considered for the studies were <50, and most of the studies were reported by researchers working in Asia. These factors also result in heterogeneity. Second, we included retrospective studies to conduct our studies, and prospective studies were not considered. In retrospective studies, it is difficult to avoid selection bias. It is also difficult to determine the time sequence of exposure and disease. The authenticity of information is debatable, and all these factors affect the accuracy of the final results. Third, it is better to establish a unified method using the DWI method as the differences in the imaging parameters, fluctuations in the field strength, and differences in the efficiencies of the post-processing software used may result in the generation of inaccurate ADC values. Thus, the result can potentially be affected.

## Conclusion

The results obtained by conducting the meta-analysis reveal that the DWI method is characterized by high sensitivity, specificity, and diagnostic accuracy. This method can be effectively used to distinguish between PCNSL and HGG. It is suggested that samples of patients suffering from PCNSL and HGG should be analyzed using the DWI to effectively distinguish between the two types of tumors as soon as possible. Further research on the diagnostic performance of DWI requires more well-designed and prospective studies involving a larger number of patients.

## Data Availability Statement

The original contributions presented in the study are included in the article/Supplementary Material, further inquiries can be directed to the corresponding author.

## Author Contributions

XD and YH conceived and designed the study, acquired and analyzed the data, and wrote the manuscript. WL contributed to data analysis and manuscript preparation. All authors read and approved the manuscript and agree to be accountable for all aspects of the research in ensuring that the accuracy or integrity of any part of the work is appropriately investigated and resolved.

## Conflict of Interest

The authors declare that the research was conducted in the absence of any commercial or financial relationships that could be construed as a potential conflict of interest.

## Publisher's Note

All claims expressed in this article are solely those of the authors and do not necessarily represent those of their affiliated organizations, or those of the publisher, the editors and the reviewers. Any product that may be evaluated in this article, or claim that may be made by its manufacturer, is not guaranteed or endorsed by the publisher.

## Supplementary Material

Supplementary Figure 1 A funnel plot representing the publication bias.

Supplementary Table 1 The results of study quality and bias judged by the Newcastle-Ottawa Scale (NOS) checklist.
