# Supplementary figures and images for: Diagnostic Accuracy of the Diffusion-Weighted Imaging Method Used in Association With the Apparent Diffusion Coefficient for Differentiating Between Primary Central Nervous System Lymphoma and High-Grade Glioma: Systematic Review and Meta-Analysis
Source: Front Neurol. 2022 Jun 24;13:882334. doi: 10.3389/fneur.2022.882334 (PMC9263097; doi:10.3389/fneur.2022.882334)

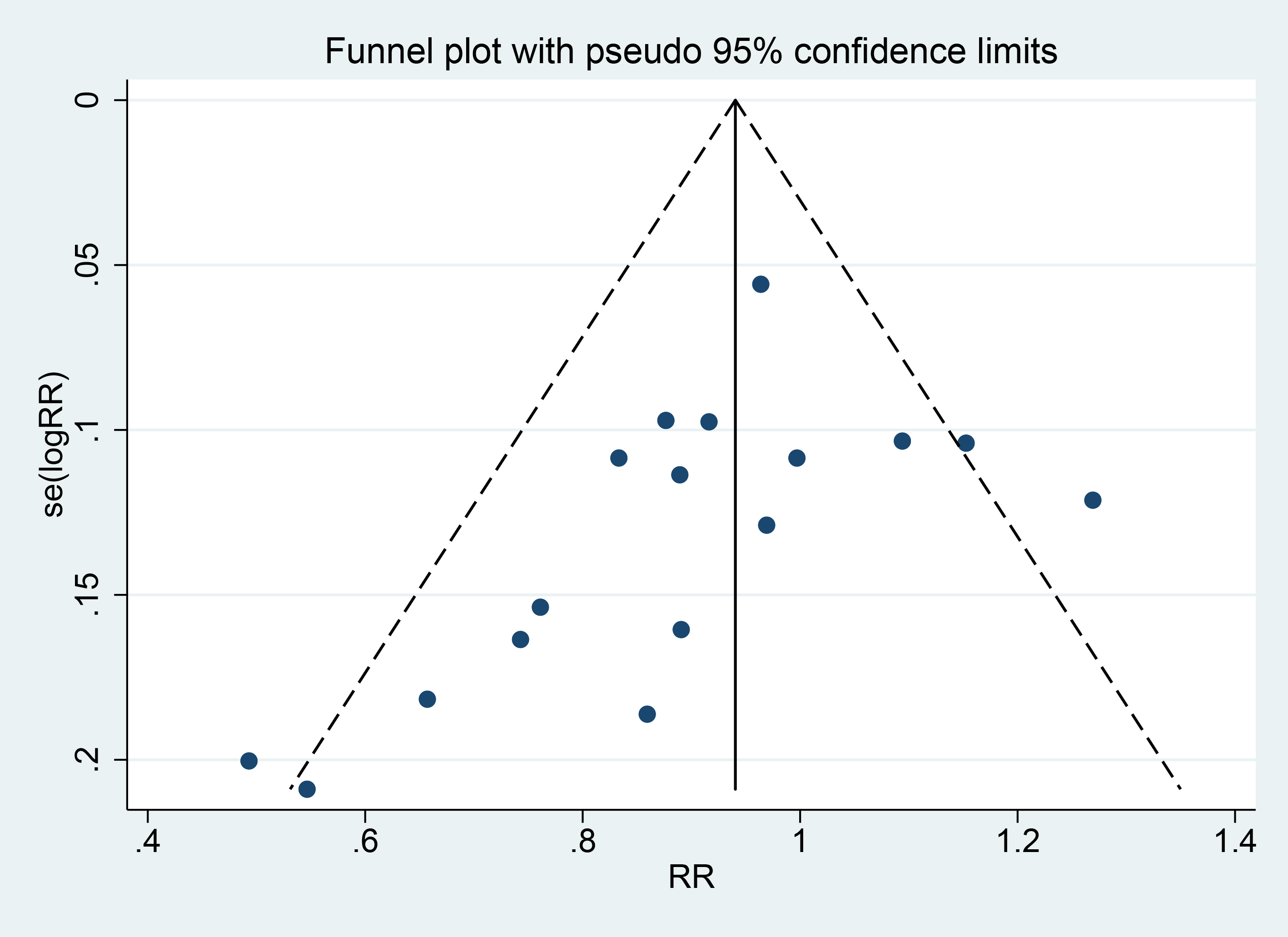

Supplement: Supplementary Figure 1 — A funnel plot representing the publication bias. [file Image_1.JPEG]
