# Supplementary material for: Diagnostic Accuracy of the Diffusion-Weighted Imaging Method Used in Association With the Apparent Diffusion Coefficient for Differentiating Between Primary Central Nervous System Lymphoma and High-Grade Glioma: Systematic Review and Meta-Analysis
Source: Front Neurol. 2022 Jun 24;13:882334. doi: 10.3389/fneur.2022.882334 (PMC9263097; doi:10.3389/fneur.2022.882334)
Supplement: Supplementary Table 1 — The results of study quality and bias judged by the Newcastle-Ottawa Scale (NOS) checklist. [file Table_1.DOC]

**Supplementary table 1.** The results of study quality and bias judged by the Newcastle-Ottawa Scale (NOS) checklist

| First author | Year | Total score | Cohort selection | | | | Comparability | Outcome | | |
| --- | --- | --- | --- | --- | --- | --- | --- | --- | --- | --- |
|  |  |  | Representativeness of the Exposed Cohort | Selection of the Non-Exposed Cohort | Ascertainment of Exposure | Demonstration that outcome of interest was not present at start of study | Comparability of cohorts on the basis of the design or analysis | Assessment of outcome | Was follow-up long enough for outcomes to occur | Adequacy of follow up of cohorts |
| Toh CH | 2008 | 7 |  | ★ | ★ | ★ | ★ | ★ | ★ | ★ |
| Doskaliyev A | 2012 | 8 | ★ | ★ | ★ | ★ | ★ | ★ | ★ | ★ |
| Yamashita K | 2013 | 7 | ★ | ★ | ★ | ★ |  | ★ | ★ | ★ |
| Ahn SJ | 2014 | 8 | ★ | ★ | ★ | ★ | ★ | ★ | ★ | ★ |
| Nakajima S | 2015 | 7 |  | ★ | ★ | ★ | ★ | ★ | ★ | ★ |
| Ko CC | 2016 | 8 | ★ | ★ | ★ | ★ | ★ | ★ | ★ | ★ |
| Li D | 2017 | 8 | ★ | ★ | ★ | ★ | ★ | ★ | ★ | ★ |
| Lin X | 2017 | 8 | ★ | ★ | ★ | ★ | ★ | ★ | ★ | ★ |
| Lu S | 2017 | 7 |  | ★ | ★ | ★ | ★ | ★ | ★ | ★ |
| Luo L | 2018 | 8 | ★ | ★ | ★ | ★ | ★ | ★ | ★ | ★ |
| Xue X | 2019 | 8 | ★ | ★ | ★ | ★ | ★ | ★ | ★ | ★ |
| Anwar SSM | 2019 | 8 | ★ | ★ | ★ | ★ | ★ | ★ | ★ | ★ |
| Eisenhut F | 2020 | 8 | ★ | ★ | ★ | ★ | ★ | ★ | ★ | ★ |
| Mehrnahad M | 2020 | 7 |  | ★ | ★ | ★ |  | ★ | ★ | ★ |
| Geng L | 2021 | 8 | ★ | ★ | ★ | ★ | ★ | ★ | ★ | ★ |
| Eyüboğlu İ | 2021 | 8 | ★ | ★ | ★ | ★ |  | ★ | ★ | ★ |
| Ozturk K | 2021 | 8 | ★ | ★ | ★ | ★ | ★ | ★ | ★ | ★ |
| Zhang S | 2022 | 8 | ★ | ★ | ★ | ★ | ★ | ★ | ★ | ★ |
